# Clinical characteristics of emergency department heart failure patients initially diagnosed as non-heart failure

**DOI:** 10.1186/1471-227X-6-11

**Published:** 2006-11-14

**Authors:** Sean P Collins, Christopher J Lindsell, W Frank Peacock, Daniel C Eckert, Jeff Askew, Alan B Storrow

**Affiliations:** 1 Department of Emergency Medicine, University of Cincinnati, Cincinnati, USA; 2 Department of Emergency Medicine, The Cleveland Clinic, Cleveland, USA; 3 Department of Internal Medicine, Division of Cardiology, University of Cincinnati, Cincinnati, USA; 4 Department of Emergency Medicine, Vanderbilt University, Nashville, USA

## Abstract

**Background:**

Since previous studies suggest the emergency department (ED) misdiagnosis rate of heart failure is 10–20% we sought to describe the characteristics of ED patients misdiagnosed as non-decompensated heart failure in the ED.

**Methods:**

We analyzed a prospective convenience sample of 439 patients at 4 emergency departments who presented with signs or symptoms of decompensated heart failure. Patients with a cardiology criterion standard diagnosis of decompensated heart failure and an ED diagnosis of decompensated heart failure were compared to patients with a criterion standard of decompensated heart failure but no ED diagnosis of decompensated heart failure. Two senior cardiology fellows retrospectively determined the patient's heart failure status during their acute ED presentation. The Mann-Whitney u-test for two groups, the Kruskall-Wallis test for multiple groups, or Chi-square tests, were used as appropriate.

**Results:**

There were 173 (39.4%) patients with a criterion standard diagnosis of decompensated heart failure. Among those with this criterion standard diagnosis of decompensated heart failure, discordant patients without an ED diagnosis of decompensated heart failure (n = 58) were more likely to have a history of COPD (p = 0.017), less likely to have a previous history of heart failure (p = 0.014), and less likely to have an elevated b-type natriuretic peptide (BNP) level (median 518 vs 764 pg/ml; p = 0.038) than those who were given a concordant ED diagnosis of decompensated heart failure. BNP levels were higher in those with a criterion standard diagnosis of decompensated heart failure than in those without a criterion standard diagnosis (median 657 vs 62.7 pg/ml). However, 34.6% of patients with decompensated heart failure had BNP levels in the normal (<100 pg/ml; 6.1%) or indeterminate range (100–500 pg/ml; 28.5%).

**Conclusion:**

We found the ED diagnoses of decompensated heart failure to be discordant with the criterion standard in 14.3% of patients, the vast majority of which were due to a failure to diagnose heart failure when it was present. Patients with a previous history of COPD, without a previous history of heart failure and with lower BNP levels were more likely to have an ED misdiagnosis of non-decompensated heart failure. Readily available, accurate, objective ED tests are needed to improve the early diagnosis of decompensated heart failure in ED patients.

## Background

Over 80% of heart failure (HF) inpatient admissions originate in the emergency department (ED). Previous studies have suggested an ED misdiagnosis rate of 10–20% [1,2]. Despite this high rate of discordance between the ED diagnosis and true diagnosis, the characteristics of those HF patients that are not initially diagnosed with heart failure in the ED have not been rigorously evaluated. We describe the clinical characteristics of HF patients not initially diagnosed as decompensated heart failure in the ED.

## Methods

### Study Design and Setting

We enrolled a prospective convenience sample of patients at 4 emergency departments who presented with signs or symptoms of decompensated HF between September 2003 and June 2004. This is a secondary analysis of a study described in detail previously [3,4]. Patients were enrolled by clinical study assistants (CSAs) at 4 urban EDs (patient volume ranging from 35,000 to 85,000 visits), 2 of which were academic departments with active residency programs and 2 of which were community centers with ED residents rotating through the ED. Briefly, patients were identified as potential participants for an electronic heart sound data investigation if they were over 18 years of age, had an electrocardiogram (ECG) ordered, had signs or symptoms of heart failure (dyspnea, lower extremity edema, fatigue, jugular venous distention, or orthopnea), and had provided written informed consent. Patients were excluded if an ECG had been performed and more than one hour had passed since they had received vasodilators or diuretics. Institutional Review Board approval was obtained at all participating sites.

### Methods of Measurement

On completion of enrollment, clinical study assistants collected demographics, past medical history, and electronic heart sound data (Audicor, Inovise Medical, Portland, OR) [5,6]. Prior to receiving laboratory or radiology results, the treating physician, blinded to electronic heart sound data, documented the presence or absence of jugular venous distension, lower extremity edema, and an S3 or S4 detected by auscultation prior to receiving laboratory and radiology results. Chest radiography, as interpreted by radiology staff, laboratory variables, b-type natriuretic peptide (BNP) levels (all 4 centers used the Triage BNP meter, Biosite, Inc, San Diego, California), treating physician ECG interpretation, in-hospital data, and in-hospital events were collected by protocol chart review performed by a single study nurse. Chart review used a standardized data collection form with predetermined data definitions. Thirty-day follow-up information was obtained by telephone interview. The death registry (Social Security Administration Death Master File Online Service) and medical records were also reviewed for all patients. Data were double entered into an electronic database for subsequent analysis.

### Criterion standard for heart failure

On completion of all data collection, and nine months after the final patient follow-up was completed, the entire medical record from the index visit for each enrolled patient was copied. All heart sound data and BNP values were removed. Two senior cardiology fellows determined the patient's HF status during their acute ED presentation. Information available to the fellows included the entire medical record from the ED and inpatient stay including ancillary testing and laboratory results, except for BNP. The heart failure fellows did not have a pre-defined set of diagnostic heart failure criteria, but were asked to make a clinical judgment regarding the etiology of each subject's symptoms based on the information available to them. Heart failure status was defined as primary acute decompensated heart failure (Primary HF) and non-heart failure (Non-HF). Primary HF was defined as presentation to the ED with acutely decompensated HF. Non-Primary HF was determined to occur when a patient was judged not to have Primary HF. If the cardiologist's reviews were discordant, the diagnosis was adjudicated by the principal investigator after reviewing the discrepant chart and having a formal meeting with both reviewers.

### Primary Data Analysis

Patients with a cardiology criterion standard diagnosis of Primary HF and an ED diagnosis of Primary HF were compared to patients with a criterion standard of Primary HF and an ED diagnosis of non-Primary HF. Further, those patients that received a diuretic in the ED, suggestive of a co-primary diagnosis of Primary HF [7], were included in the ED Primary HF group and a similar comparison was performed. Data are described using medians and ranges for continuous data, and frequencies and percents for categorical data. The Mann-Whitney U-test for two groups, the Kruskall-Wallis test for multiple groups, Chi-square tests, or Fisher's Exact tests were used as appropriate. Analyses were performed using SPSS v13.0 (SPSS Inc, Chicago, IL) and Microsoft Excel (Microsoft Corporation, Redmond, WA).

## Results

### Characteristics of study subjects

There were 439 subjects enrolled; all subjects were included in this analysis. The median age was 61, 52.4% were female, and 49.2% were white. There was a prior diagnosis of HF in 50.1% of patients (Table 1). There were 173 (39.4%) patients with a criterion standard diagnosis of Primary HF and 266 (60.6%) patients with non-Primary HF. The reviewers agreed 86.0% of the time on the presence of Primary HF (kappa = 0.77). In cases where there was a disagreement (n = 43), the case was adjudicated as Primary HF in 58.1% (n = 25) and non-Primary HF in 41.9% (n = 18).

**Table 1:** Clinical characteristics and test results for all enrolled subjects, stratified by criterion standard diagnosis of Primary versus non-Primary heart failure. Data are given as means and standard deviations or as frequencies and percents.

	**Non-Primary HF N = 266 (60.6%)**		**Primary HF N = 173 (39.4%)**		**Total N = 439**		**p-value**
**Age**	58.5	(15.4)	66.7	(15.7)	61.6	(16.0)	<0.001
**Male**	116	(43.6)	93	(53.8)	209	(47.6)	
**Female**	150	(56.4)	80	(46.2)	230	(52.4)	0.040
**Non-white**	130	(48.9)	93	(53.8)	223	(50.8)	
**White**	136	(51.1)	80	(46.2)	216	(49.2)	0.330
**History of CHF**	74	(27.8)	146	(84.4)	220	(50.1)	<0.001
**History of CAD**	76	(28.6)	80	(46.2)	156	(35.5)	<0.001
**History of hypertension**	144	(54.1)	132	(76.3)	276	(62.9)	<0.001
**History of valvular heart disease**	48	(18.0)	82	(47.4)	130	(29.6)	<0.001
**History of COPD**	40	(15.0)	28	(16.2)	68	(15.5)	0.788
**CHF admission in last 6 months**	19	(7.1)	45	(26.2)	64	(14.6)	<0.001
**Prior EF <55%**	9	(18.0)	33	(60.0)	42	(40.0)	<0.001
Median BNP level*	62.7	(5–5000)	657	(5–5000)	278	(5–5000)	<0.001

*Median and range of BNP levels.

### Characteristics of patients with a criterion standard diagnosis of Primary HF

Patients with a criterion standard diagnosis of Primary HF were more likely to be male, have a previous history of heart failure, hypertension, valvular heart disease, a prior history of heart failure admissions, and a previous ejection fraction less than 55% (p < 0.05 for all comparisons) than patients without Primary HF. Median BNP levels in patients with Primary HF were 657 pg/ml compared to 62.7 pg/ml in those with non-Primary HF (p < 0.001) (Table 1). In patients with Primary HF, 10 (6.1%) had BNP levels less than 100 pg/ml, and 47 (28.5%) had BNP levels in the range of 100–500 pg/ml.

### ED Misdiagnosis Rate and Clinical Characteristics

The overall rate of discordance between the ED diagnosis and the criterion standard diagnosis was 14.3% (95% CI 11.3%-18.1%). Of these 63 cases, 58 were cases where the ED physician did not diagnose Primary HF when it was found to be present by the criterion standard (Table 2). These 58 patients were less likely to have a previous history of HF (p = 0.014) and more likely to have a previous history of COPD (p = 0.017) than those patients with both an ED diagnosis and a criterion standard diagnosis of Primary HF (n = 115). Median BNP levels in discordant patients without an ED diagnosis of Primary HF (518 pg/ml) were significantly lower than those patients with an ED Primary HF diagnosis (764 pg/ml) (p = 0.038). No other differences were found when evaluating history, physical examination and vital signs (p > 0.05, Table 3).

**Table 2:** ED Diagnosis compared with final criterion standard diagnosis.

		**Criterion Standard Diagnosis**	
		*Primary HF*	*Non-Primary HF*

**ED Diagnosis**	*Primary HF*	115 (26.3%)	**5 (1.1%)**
	*Non-Primary HF*	**58 (13.2%)**	260 (59.4%)

**Table 3:** Demographic and clinical characteristics of patients with a criterion standard diagnosis of heart failure (n = 173) stratified by ED diagnosis of heart failure.

		**ED Diagnosis**				**P-value**
		**Non-Primary HF N = 58**		**Primary HF N = 115**		
**Age**		66	(34–88)	70	(30–97)	0.318
**Non-white**		32	(55.2)	61	(53.0)	0.872
**White**		26	(44.8)	54	(47.0)	
**Male**		32	(55.2)	61	(53.0)	0.872
**Female**		26	(44.8)	54	(47.0)	
**History of CHF**		43	(74.1)	103	(89.6)	0.014
**History of CAD**		21	(36.2)	59	(51.3)	0.076
**History of hypertension**		47	(81.0)	85	(73.9)	0.347
**History of valvular heart disease**		22	(37.9)	60	(52.2)	0.106
**History: cardiomyopathy**		20	(34.5)	44	(38.3)	0.739
**History of COPD**		15	(25.9)	13	(11.3)	0.017
**CHF admission in last 6 months**		16	(27.6)	29	(25.4)	0.855
**Prior EF <55%**		11	(55.0)	22	(62.9)	0.582
**Positive findings on CXR**		47	(81.0)	89	(77.4)	0.696
**Systolic blood pressure**		144	(82–234)	152	(85–257)	0.915
**Diastolic blood pressure**		81	(39–140)	84	(40–167)	0.751
**Heart rate**		95	(52–158)	88	(44–142)	0.177
**Respiration rate**		20	(12–44)	22	(12–52)	0.481
**Oxygen saturation**		95	(72–100)	96	(60–100)	0.959
**Temperature**		97.4	(95.4–101.1)	97.5	(695.0-101.2)	0.848
**Symptoms of jugular venous distention**		19	(33.3)	39	(33.9)	1.000
**Symptoms of peripheral edema**		35	(61.4)	74	(64.9)	0.736
**Symptoms of dyspnea**		54	(93.1)	112	(97.4)	0.226
**Symptoms of orthopnea**		38	(65.5)	73	(63.5)	0.867
**Symptoms of proxysmal nocturnal dyspnea**		22	(38.6)	43	(37.7)	1.000
**LVH**		16	(31.4)	34	(33.7)	0.856
**QRS > 120 ms**		15	(29.4)	38	(37.6)	0.370
**Left Bundle Branch Block**		1	(2.0)	8	(7.9)	0.273
**Physician identified S3**		8	(14.3)	22	(19.6)	0.522
**Electronically detected S3**		15	(34.1)	30	(33.7)	1.000
**Median BNP**		518	(5–5000)	764	(31.7–5000)	0.038
**Ejection Fraction**	**Normal**	4	(19.0)	9	(14.8)	0.059
	**Mild**	9	(42.9)	18	(29.5)	
	**Moderate**	6	(28.6)	10	(16.4)	
	**Severe**	2	(9.5)	24	(39.3)	

### ED Misdiagnosis Rate when diuretic use was considered

Further, those patients that received a diuretic in the ED, suggestive of a co-primary diagnosis of Primary HF [7], were included in the ED Primary HF group and a similar comparison was performed. When patients that received diuretics in the ED were included in the ED Primary HF diagnostic category the number of discordant diagnoses decreased from 14.3 % to 10.7% (5.7% misdiagnosed as non-Primary HF and 5.0% misdiagnosed as Primary HF). Patients with an ED diagnosis of non-Primary HF were less likely to have a prior history of heart failure and more likely to have intermediate BNP levels (median 447.0 pg/ml in patients with a discordant ED diagnosis vs. 758.0 pg/ml in patients with a concordant ED diagnosis) than those patients with a concordant ED diagnosis of Primary HF (p = 0.006 and p = 0.024, respectively). None of the other demographics, vitals signs or laboratory parameters was significantly different between those with ED discordant and concordant Primary HF diagnoses (p > 0.05.)

## Discussion

Consistent with previous studies we found an ED discordant diagnosis rate of 14.3% [2,8]. The vast majority of these discordant diagnoses were due to a failure to diagnose Primary HF when it was present. Characteristics of these patients with a discordant ED diagnosis of non-Primary HF have not been previously well described. Patients with a discordant ED diagnosis of non-Primary HF were less likely to have a previous history of heart failure and an elevated BNP level compared to those with a concordant Primary HF diagnosis. These results remained significant even when patients that received diuretics in the ED were included as Primary HF.

Our previous findings in this clinical study were that the prior administration of diuretics and vasodilators may affect heart sounds findings [9], and that the combined use of BNP and an S3 improves diagnostic accuracy in ED patients with heart failure [4]. In conducting those analyses, a discrepancy was noted between the ED physician's diagnosis and the criterion standard, a phenomenon which has also been observed in other studies [1,2]. In this study, therefore, we explicate the discordance between the ED diagnosis and the criterion standard by describing the differences and similarities between patients with discordant and concordant diagnoses. As has been reported previously, physical examination findings such as jugular venous distension, peripheral edema, and an S3 heart sound were not helpful for establishing a diagnosis of Primary HF [10,11]. While patients with an ED diagnosis of Primary HF had higher overall median BNP levels than HF patients without an ED diagnosis of Primary HF, over one-third of patients had BNP levels that were either considered normal (<100 pg/ml) or in the indeterminate range of 100–500 pg/ml. While BNP has been suggested to be useful to "rule-out" Primary HF when it is <100 pg/ml, our findings suggest its use in the indeterminate zone continues to be problematic [8]. Further, these findings also suggest that patients who have intermediate BNP levels may benefit from further testing before a diagnosis of Primary HF can be either confirmed or rejected. Other studies have suggested that age, sex and race influence BNP levels, and that BNP may be best utilized at two different cutoff levels: one that "rules out" Primary HF (<100 pg/ml) and one that "rules in" Primary HF (>500 pg/ml) [12-14].

Congestion on chest radiography was present in 81.0% of HF patients without an ED Primary HF diagnosis. While congestion on chest radiography is often considered diagnostic for Primary HF, there are several possibilities why patients with these findings were felt to not have Primary HF by the ED physician. An official radiographic interpretation (board certified staff radiologist) may not have been available at the time of ED evaluation, or perhaps the preliminary reading did not correlate with the official interpretation (which was used in this analysis and during determination of the criterion standard). Also, other findings on chest radiography (focal air space disease) or ancillary tests (renal function, urinalysis) may have suggested that an alternative primary diagnosis was present along with Primary HF.

### Limitations

The use of independent blinded reviewers to determine the criterion standard diagnosis is currently considered a useful, objective process [8,15]. However, there are some limitations with this method. The entire clinical course of the patient is not always available in the medical record and omissions regarding response to therapy along with the advantage of being able to follow a patient clinically during their ED stay may have a significant impact on diagnosis. A better, yet much more labor intense criterion standard, would be to have an independent reviewer follow the patient in the ED and while in the hospital. This would facilitate a diagnosis based on response to therapy, further diagnostic testing, and change in physical examination findings.

Emergency physicians were not blinded to BNP levels during the study. BNP levels were considered part of the standard care work-up in this cohort. The investigators did not feel it was ethical to withhold this information from the treating physician. This may have introduced incorporation bias due to the influence of BNP levels on the treating physician's diagnosis.

Given the relative paucity of definitive diagnostic tests available, an ED diagnosis may be more general (dyspnea NOS) than specific (Primary HF). The treating emergency physician was not required to record their diagnosis of primary versus non-primary heart failure in a dichotomous manner. This may have led to bias that resulted in those patients that were given a non-specific diagnosis (i.e. dyspnea NOS) being counted as non-Primary HF when the treating physician may have felt the contrary. While treating physicians may have felt that patients had a component of Primary HF (evidenced by the administration of diuretics, which occurs in 95% of ED patients with Primary HF [7]) they may have been reluctant to label the patient as "Primary HF" until left ventricular dysfunction was confirmed by further testing. The authors attempted to correct for this bias by determining those non-Primary HF patients that had received diuretics in the ED and re-analyzing the data. Including those patients that had received diuretics in the ED as "Primary HF" minimizes this potential bias. This analysis suggests that, while the number of discordant diagnoses decreased from 14.3% to 10.7%, the characteristics (lack of a previous history of HF and intermediate BNP levels) of patients with discordant diagnoses did not change.

When determining final chest radiograph interpretations, staff radiologists were not blinded to clinical information. The consistency of congestion on chest radiographs may reflect incorporation bias on the part of the attending radiologist. A true measure of congestion on chest radiograph would only be determined if staff radiologists were blinded to clinical information.

## Conclusion

In this study of ED patients with suspected heart failure we found the ED diagnoses to be discordant with the criterion standard in 14.3% of patients. The vast majority of these were due to a failure to diagnose Primary HF when it was present. Patients with a previous history of COPD and without a previous history of heart failure were more likely to have an ED misdiagnosis of non-Primary HF. BNP levels did not reliably differentiate Primary HF from non-Primary HF in the ED. Readily available, accurate, objective ED tests are needed to improve the early diagnosis of Primary HF in the ED.

## Competing interests

The original data collection for the database that was used for this secondary analysis was paid for by Inovise Medical, Inc.

Dr. Collins- has received research support and honoraria from Inovise Medical, Inc

Dr. Lindsell- has received research support from Inovise Medical, Inc.

Dr. Peacock- has received research support and is a consultant for Inovise Medical, Inc and received research support and honoraria from Biosite, Inc.

Dr. Askew- has no competing interests

Dr. Eckert- has no competing interests

Dr. Storrow- has received research support from Inovise Medical, Inc and Biosite, Inc. and honoraria from Biosite, Inc

## Authors' contributions

AS, FP, CL, SC designed the original study. SC, DE, JA, CL participated in database generation, cleaning and analysis. AS, FP, CL, DE, JA, SC all contributed to manuscript preparation and have given final approval to the submitted manuscript.

## Pre-publication history

The pre-publication history for this paper can be accessed here:


